# Successful Percutaneous Coronary Intervention in a Patient With aVR ST-Segment Elevation Myocardial Infarction Due to Spontaneous Atherosclerotic Coronary Artery Dissection

**DOI:** 10.7759/cureus.19545

**Published:** 2021-11-13

**Authors:** Yudistira Santosa, Angelina Yuwono

**Affiliations:** 1 Internal Medicine, Atma Jaya Catholic University of Indonesia, Jakarta, IDN

**Keywords:** cardiology, vascular, coronary angiography, percutaneous coronary intervention, pci, primary pci, sudden atherosclerotic coronary artery dissection, scad, myocardial infarction, stemi

## Abstract

Chest pain is a common clinical symptom that leads to a patient's admission to the emergency department, which may be caused by acute coronary syndrome (ACS). Electrocardiography (ECG) is a useful tool for diagnosis, risk stratification, and treatment response monitoring in clinical practice. The coronary angiography should be done in ACS, which may detect spontaneous atherosclerotic coronary artery dissection (SCAD) that should be followed by urgent revascularization. We present a case of a 55-year-old male with the augmented Vector Right (aVR) ST-segment elevation myocardial infarction due to spontaneous atherosclerotic coronary artery dissection. The patient had a good outcome after we performed early coronary angiography, followed by the percutaneous coronary intervention (PCI).

## Introduction

Acute chest pain is the subjective feeling of chest discomfort that occurs within 24 hours and encompasses various qualities of perceptions [1]. Acute chest pain is one of the most common symptoms that leads to the patient’s admission to the emergency department (ED) and may be a sign of a life-threatening condition [1-2]. Twenty-five percent (25%) of patients with acute chest pain are diagnosed with acute coronary syndrome (ACS) [1]. A recording and interpretation of an ECG and examination of blood cardiac biomarkers should be done within 10 minutes of admission for patients with acute chest pain [1]. ST-segment elevation (STE) in the augmented Vector Right (aVR) lead is a useful tool in recognizing patients with a left main or left anterior descending coronary obstruction during acute coronary syndrome (ACS) [3].

Patients with spontaneous coronary artery dissection (SCAD) usually show symptoms and signs similar to acute myocardial infarction [4-5]. SCAD is defined as a spontaneous separation of the inner intimal lining from the outer vessel wall due to atherosclerotic coronary artery disease and non-atherosclerotic variant [5]. Hereby, we reported a case of a 55-year-old male who was admitted to the ED for acute chest pain. Further evaluation showed aVR STE myocardial infarction due to spontaneous atherosclerotic coronary artery dissection. This case report aims to increase our awareness of SCAD in patients presenting with chest pain and how it should be managed.

## Case presentation

A 55-year-old, male, active smoker, came with acute onset chest pain one hour before admission. The chest pain was central, dull, heavy, and radiated to the left arm. The pain occurred when he walked out of his bedroom and was not relieved by sublingual nitrate or rest. The chest pain was associated with shortness of breath. He had begun to feel intermittent chest pain in the past two months and he had been evaluated with an echocardiogram, which showed normal ejection fraction (EF 60.9%), and a multi-slice CT angiogram, which revealed three-vessel and left main (LM) disease (moderate stenosis in LM severe stenosis, in osteal left circumflex (LCx), and moderate stenosis in osteal-mid left anterior descendent (LAD), moderate to severe stenosis in mid to distal right coronary artery (RCA)). He denied any history of hypertension, diabetes mellitus (DM), or renal disease. He had no family history of a similar disease and had no history of surgery or trauma around his chest.

**Figure 1:**
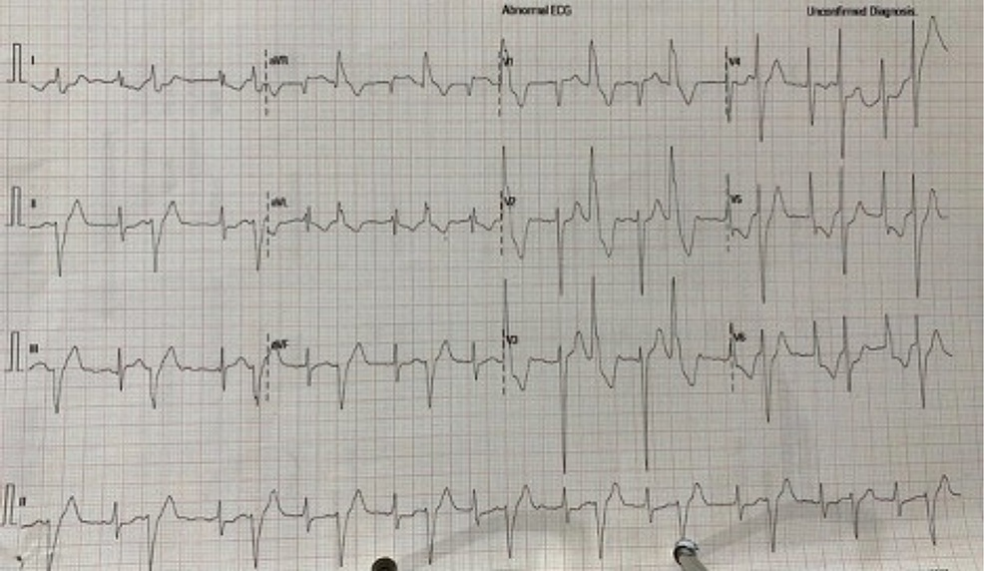
Electrocardiogram (ST elevation in aVR lead, ST depression in V5-V6 with VES Bigeminy)

On admission, the patient was alert but lethargic. His vital signs on admission showed blood pressure 100/60 mmHg, an irregular tachyarrhythmia (heart rate 120 beats/minute), and tachypnoea (respiratory rate 28 breaths/minute). We found normal heart sounds without any murmur or additional sounds. His pulmonary examination was unremarkable. His laboratory workup on admission showed normal complete blood count, renal function, and normal blood glucose level. He had elevated troponin I level of 159.4 ng/mL (normal < 2 ng/mL). The initial ECG showed ST elevations in the aVR lead and ST depression in anterolateral leads with bigeminy ventricular extrasystole (VES) (Figure 1). 

**Figure 2:**
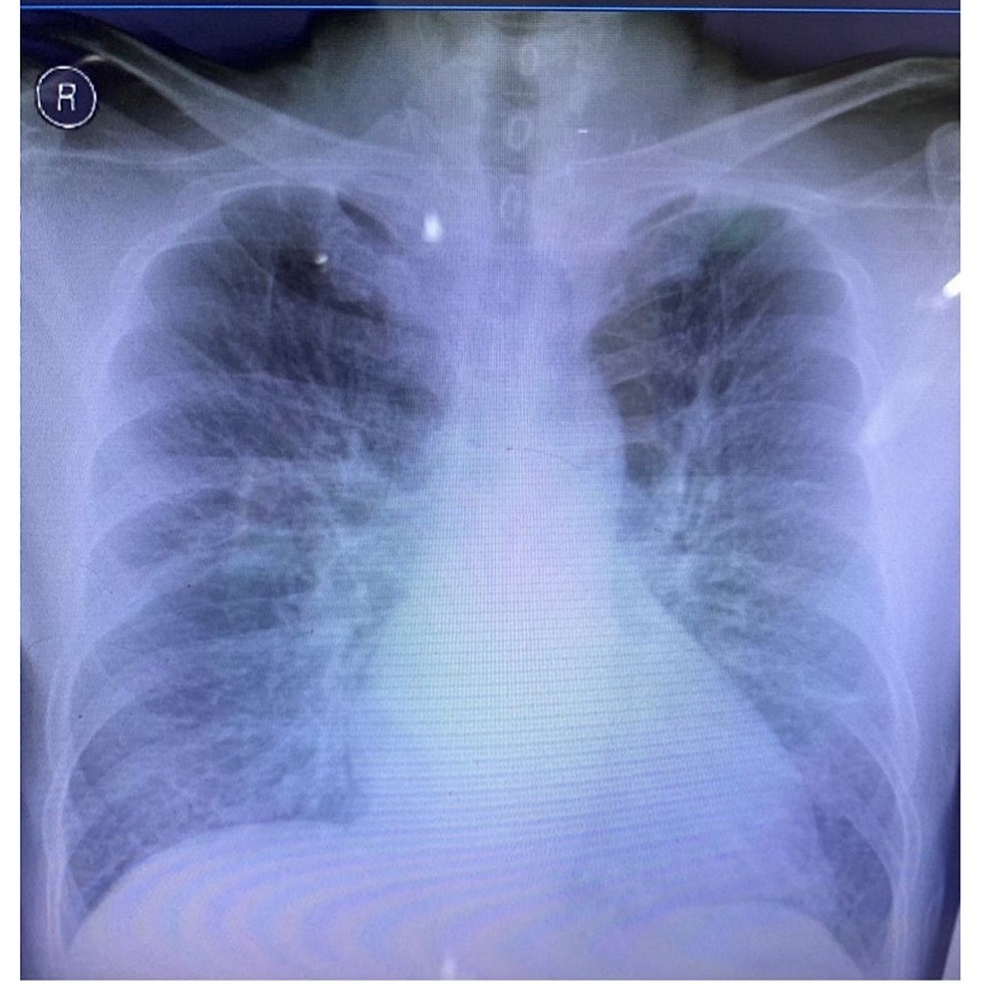
Chest X-ray showed cardiomegaly and acute lung edema

Chest X-ray revealed cardiomegaly and impending acute lung edema (Figure 2).

**Figure 3:**
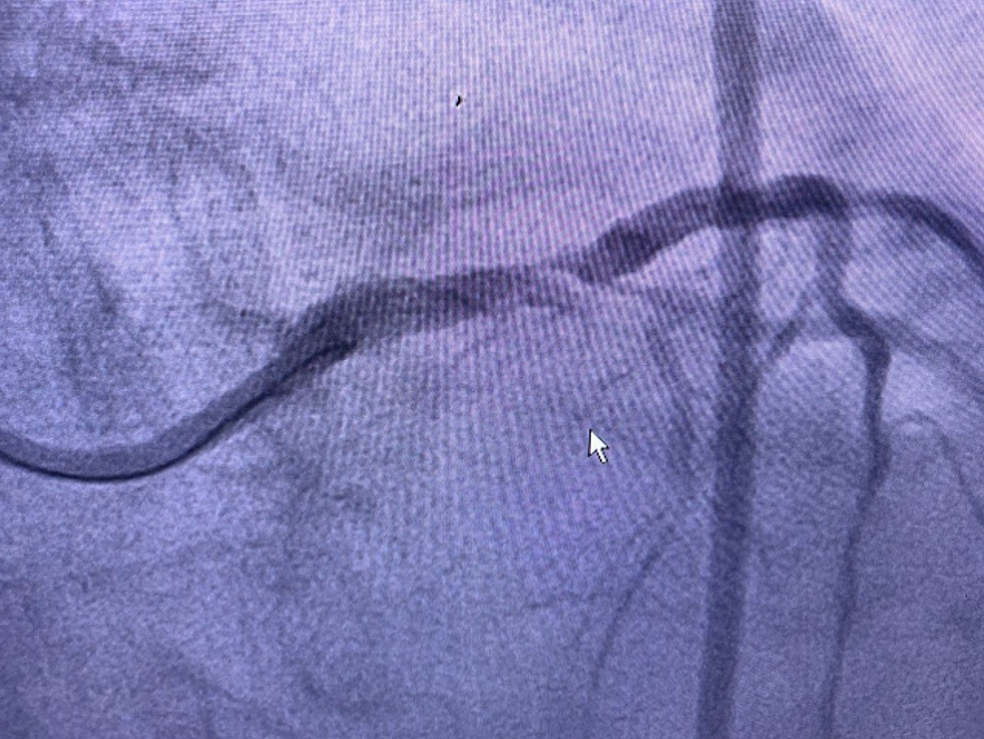
Dissection left main-left circumflex (LM-LCx)

**Figure 4:**
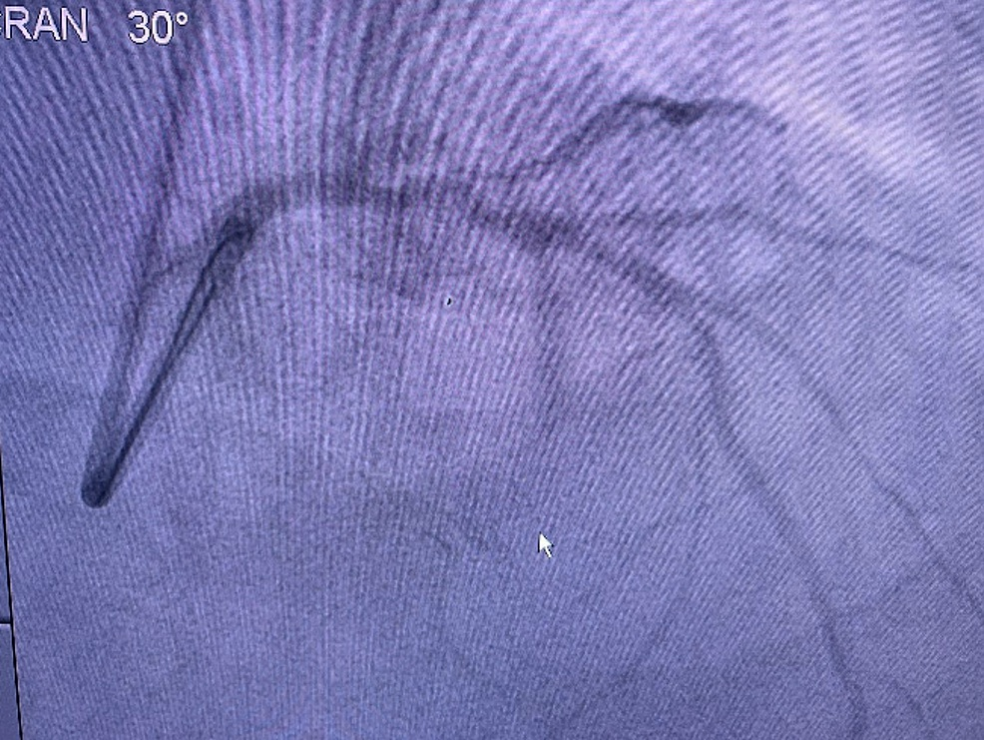
Non-significant stenosis in LAD LAD: left anterior descendent

**Figure 5:**
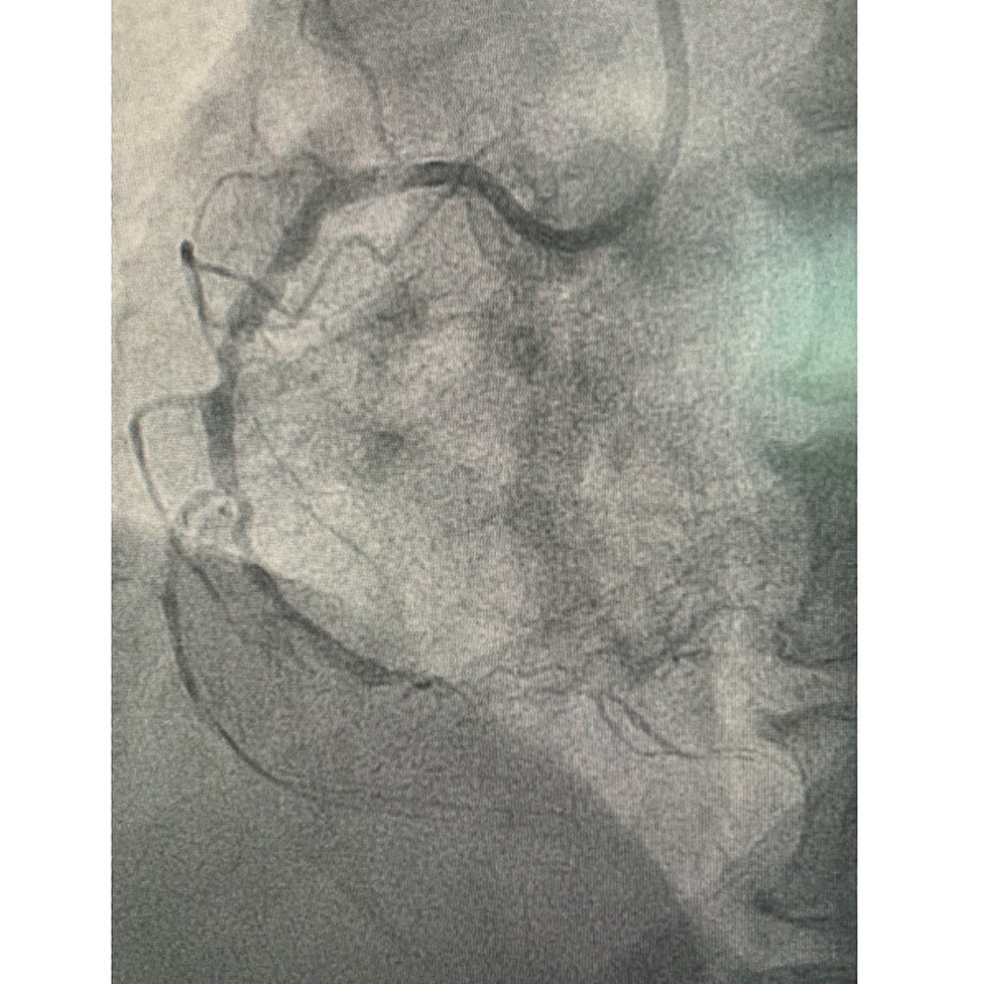
Right coronary artery (RCA) angiogram

The patient was diagnosed by an aVR ST-elevation myocardial infarction (STEMI) with bigeminy VES and impending acute lung edema. The patient was given oxygen supplementation, a loading dose of ticagrelor and aspirin, furosemide injection, followed by the primary percutaneous coronary intervention (PCI). Cardiac catheterization showed angiographically diffuse stenosis in the mid-segment and total stenosis in RCA, a dissection that was caused by a ruptured plaque in the left main-left circumflex (LM-LCx) portion and non-significant stenosis in the mid LAD (Figures 3-5 and Video 1).

**Video 1:** Coronary angiogram findings before stent placement

**Figure 6:**
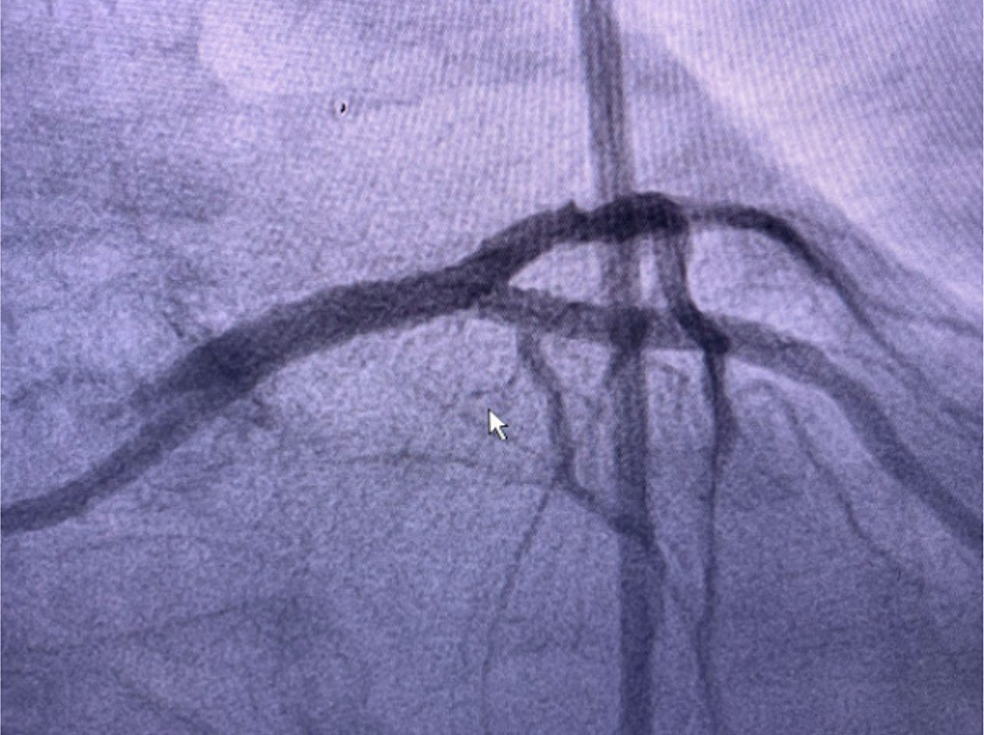
Post percutaneous coronary intervention (PCI) 1 drug-eluting stent in left main-left circumflex (LM-LCx) portion

**Figure 7:**
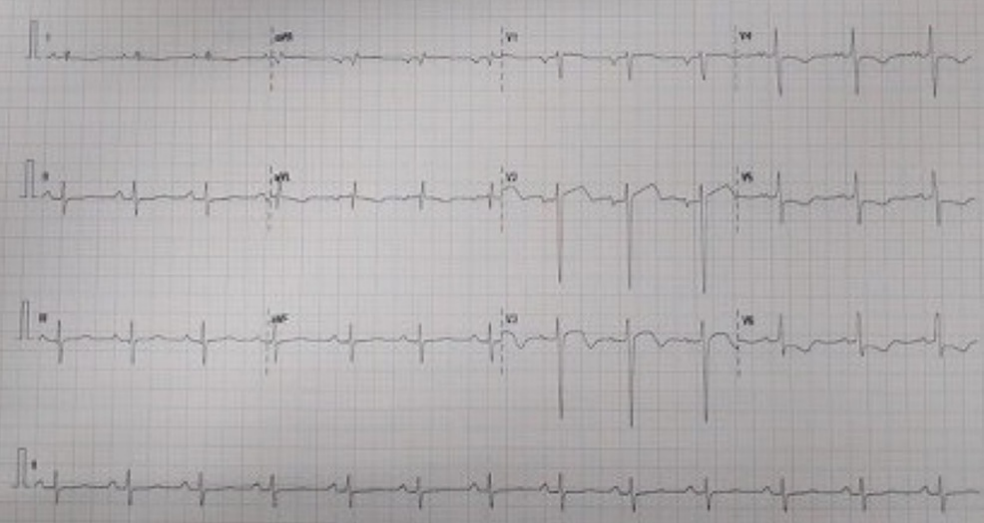
Electrocardiogram post percutaneous coronary intervention (PCI)

PCI was done with EBU 3.5/7Fr, double guidewire to LAD, and LCx portion with Crusade microcatheter guidance (Kaneka, Japan). A drug-eluting stent (stent 3.0x34 mm) was placed in the LM-LCx portion. Post that, the LM portion was dilated with a balloon of 4.0x9 mm. The process was shown in Video 2. The post PCI result is shown in Figure 6 and the electrocardiogram post PCI is shown in Figure 7.

**Video 2:** Stent placement process

**Figure 8:**
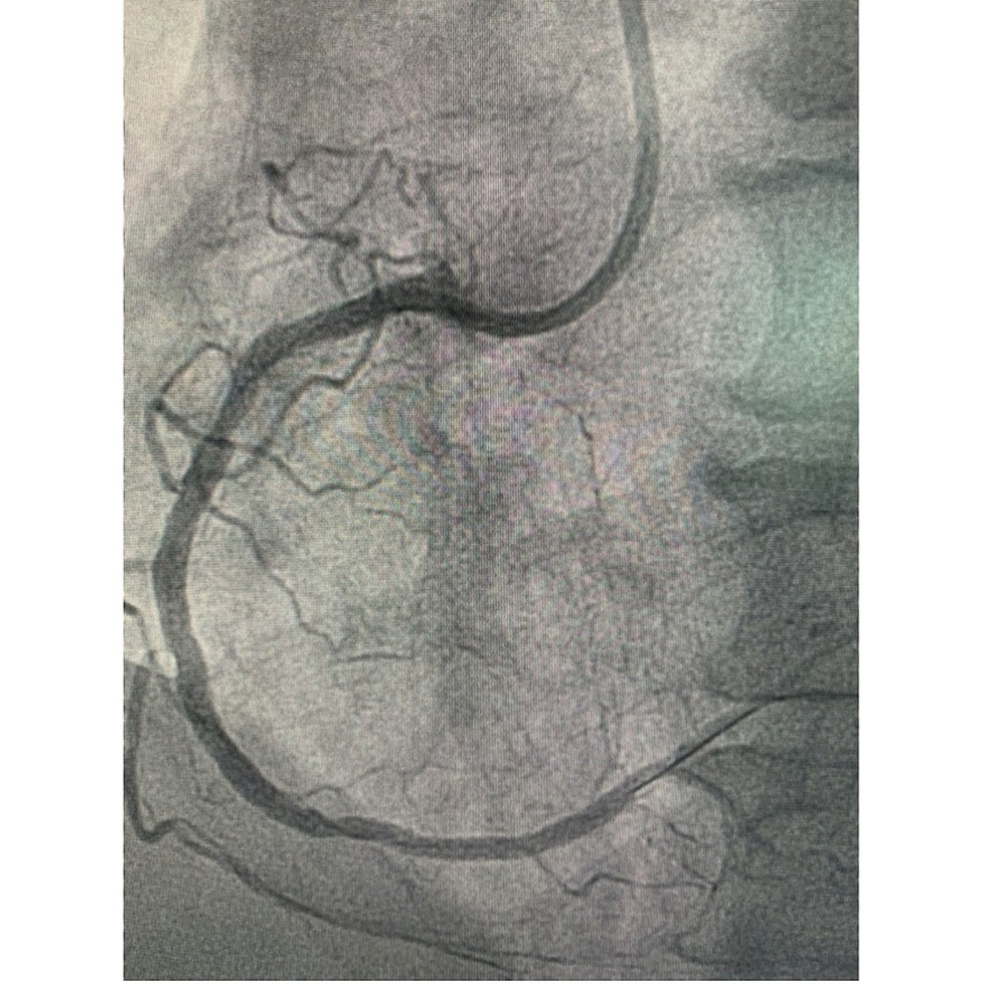
Post percutaneous coronary intervention (PCI) in right coronary artery (RCA) with two drug-eluting stents

After the PCI, he was medically optimized and hemodynamically stabilized. He was discharged after five days of hospitalization with some medication continued after being discharged, including dual antiplatelet therapy, high-intensity statin, and bisoprolol. After several weeks, RCA was treated with two drug-eluting stents. Post PCI in the RCA segment is shown in Figure 8.

## Discussion

Acute coronary syndrome (ACS) is characterized by erosion or rupture of plaques in coronary arteries. Chest pain, ECG, and cardiac biomarkers are used to distinguish myocardial infarction (MI) [2]. The ECG is a useful tool for early diagnosis, as it quickly detects ischemic changes, which is shown by the presence of ST-segment changes (depression/elevation), inverted T and Q waves changes [2,6-7]. The ECG is also used for risk stratification and for monitoring treatment response in clinical practice [6]. The augmented Vector Right (aVR) lead is unipolar and normally faces the cardiac apex to the outflow tract of the right ventricle [6]. Changes in lead aVR occur if myocardial strain and ischemia are present, such as in acute coronary syndrome, pulmonary embolism, and pericarditis. aVR ST elevation in ACS foretells poor prognosis, as it correlates with higher in-hospital mortality rate, reinfarction, heart failure, and 90-day mortality [8]. ST-elevation (STE) in lead aVR is present in patients with three-vessel disease, left main (LM) disease, acute pulmonary embolism, acute aortic dissection, and myocardial hypertrophy [9]. Our patient had typical chest pain and shortness of breath. Further examination revealed lead aVR ST-elevation and ST-depression in anterolateral leads, bigeminy VES, and elevated troponin I level (159.4 ng/mL, normal <2 ng/mL). As in the previous study, the lead aVR abnormality in this patient was due to the three vessels and left main disease, which had previously been found on his routine CT angiogram. Ventricular tachyarrhythmias (Vas) are commonly found after acute MI and are related to higher mortality risk. The VES or premature ventricular contractions (PVCs) usually occur in early ischemia [10]. Myocardial ischemia leads to mechanical strain that predisposes patients to develop VAs and to the development of an injury current, an altered current that reactivates the heart during ischemia [11]. In our patients, bigeminy VES was cleared after the ischemic lesion was treated.

SCAD is the separation of the coronary arterial walls due to non-traumatic and non-iatrogenic causes. SCAD is a rare form of ACS and mostly occurs in females [12]. It is often misdiagnosed due to a low suspicion of ACS in young women, and it is underdiagnosed due to some limitations of coronary angiography techniques and the lack of clinicians’ familiarity with the disease. Although those who had SCAD mostly have no traditional cardiovascular risk factors, some factors such as genetic factors, underlying arteriopathies, systemic inflammatory disease (connective tissue disease and/or vascular disease), and hormonal influences, may contribute to the development of SCAD [12-13]. Those factors are triggered by hormones (e.g. pregnancy), stimulants or illicit drugs, and physical and emotional stressors. Men with SCAD have a slightly younger age than women. In men, SCAD usually occurs at the right coronary artery in patients with previous coronary artery disease (CAD) or cardiovascular risk factors and may be triggered by a physical stressor (such as heavy lifting or exercise) [13]. Our patient was a male who had previous coronary artery disease, which is similar to the previous findings. In women, SCAD occurs mostly at the LAD and is observed during the peripartum and early post-partum period in those who have no cardiovascular risk factors and in the absence of CAD [4,12].

SCAD is marked by the formation of an intramural hematoma (IMH) that separates the epicardial layers of the coronary arterial wall [4,13-14]. There is a separation in the outer third of the tunica media and IMH inside the dissection [13]. It is developed due to a disruption in the vessel wall or intimal tear, which later is filled by blood and called the “false lumen.” It may also be caused by a spontaneous hemorrhage within the vessel wall. In patients with an intimal tear, we should consider whether it is due to the pressure increase in the false lumen, which causes intimal rupture into the true lumen, or it may be the initiating events, or perhaps it is the effect of coronary imaging instrumentation. Other predominant findings are fibromuscular dysplasia (FMD), eosinophilic infiltration, and so on [13]. The IMH or dissection flap may obstruct the coronary artery, cause coronary insufficiency, and further develop acute myocardial infarction [12-13].

SCAD may be the cause of ACS in 1%-4% of patients [13]. Twenty-six percent to 87% present with ST-segment-elevation MI, 13%-69% present with non ST-segment-elevation MI, and a small portion of patients develop cardiogenic shock [13]. In the most extreme cases, patients may have ventricular arrhythmia and sudden cardiac arrest as their first clinical manifestation (3%-11% patients) [4,12]. Typical angina pectoris is the most prevalent symptom of SCAD (90%) and, as in ACS, SCAD patients may have their cardiac enzyme levels elevated [13-14]. SCAD should be considered as the cause of chest pain in patients with young age who have no atherosclerotic risk factors [13]. Distinguishing SCAD and atherosclerotic acute MI need to be done as soon as possible because of the different management between the two diseases [14].

Coronary angiography is used to ensure accurate and early diagnostic of the SCAD, especially in patients with ST-segment-elevation MI, which gives the appearance of spiral dissection, extraluminal contrast staining with multiple radiolucent lumens, and intraluminal filling defects [4,13,15]. LAD is the most affected artery in SCAD (45%-61% cases), followed by the circumflex, ramus, and obtuse marginal branches in 15%-45% of cases. Cases of SCAD in the left main artery are up to 4%, and this SCAD is usually associated with STEMI [13,16]. Left main, or ostial left anterior descending artery, or multivessel severe proximal coronary artery dissection are categorized as high-risk anatomy [13-14]. SCAD is classified into three types based on the angiography finding. Those with multiple radiolucent lumens or arterial wall contrast staining are classified as type 1. Type 2 is the most common type of SCAD. These patients have diffuse stenosis, which varies in length (>20 mm) and severity, and later differentiated into variants 2A and 2B. We can find diffused arterial narrowing, which is bordered by normal proximal and distal segments to the IMH in variant 2A, and diffused narrowing, which extends to the distal artery in variant 2B. Focal or tubular stenosis (<20 mm), which is similar to atherosclerosis, is classified as type 3. Intracoronary imaging, by optical coherence tomography or intracoronary ultrasonography, aids in the diagnosis of SCAD in non-diagnostic cases. This modality allows us to detect intimal tear, false lumen, intraluminal thrombi, and IMH [13]. One non-invasive technique, which can be used for evaluating IMH in patients with an uncertain diagnosis of SCAD after catheterization (especially the proximal lesion) is coronary computed tomographic angiography (CCTA). The findings of atherosclerotic plaque on CCTA may be mistaken for an IMH, which limits the visualization of SCAD [14]. Our patients, who had typical chest pain, ischemic ECG changes with PVCs, and elevated cardiac enzyme, underwent PPCI, and during the process, we found a diffused stenosis in the mid-segment, total stenosis in RCA, and non-significant stenosis in mid LAD. Later, we found SCAD in LM-LCx and placed a drug-eluting stent.

Although they may present as ACS, SCAD patients have three major differences from patients with atherosclerotic coronary lesions. First, medial dissection is the pathophysiological hallmark of SCAD. Second, PCI is associated with worse outcomes in SCAD patients than in those with atherosclerotic lesions. Third, if it is medically treated, SCAD lesions heal over time, the stenosis is decreased and, eventually, the blood flow is restored. All patients with acute SCAD should be admitted to detect complications and recurring MI [14]. The current consensus suggests three to five days of observation in the hospital for ACS patients with SCAD [13-14].

SCAD in those who are clinically stable and have no high-risk anatomy may be treated with conservative management and should be monitored in the hospital for three to five days [13-14]. Of those who are clinically stable with high-risk anatomy, coronary artery bypass grafting (CABG) should be put into consideration [13]. The anticoagulant should be discontinued after the SCAD had been confirmed. Thrombolysis is avoided because it decreases the formation of thrombus in the false lumen and increases the blood flow in the true lumen, which eventually increases bleeding into the false lumen and contributes to the extension of SCAD [12,17]. The consumption of dual antiplatelet therapy in SCAD is under debate. Some studies showed promising results while others showed an increased risk of bleeding in these patients. Some experts recommend consuming aspirin for at least one year in SCAD patients who were medically treated if there are no contraindications. The b-blockers, angiotensin-converting enzyme inhibitors (ACEis), or angiotensin receptor blockers (ARBs) are considered in patients with ventricular dysfunctions. B-blockers should be considered in patients with arrhythmia. Statin therapy is not a routine medication after SCAD. It is given to prevent atherosclerosis and to manage the concomitant atherosclerotic disease. Anti-anginal therapy should be given to relieve chest pain in SCAD [13].

Patients with active or ongoing ischemia, as our patient, or those with unstable hemodynamic, also classified as patients with high-risk clinical features, should undergo PCI or urgent coronary artery bypass graft (CABG) [4,13-14]. Revascularization is also indicated in patients with VAs or left-main dissection, as in our patient [5,13]. PCI has to be undertaken cautiously with meticulous techniques. It may be challenging to enter the true lumen with the coronary guidewire. If this fails, emergent CABG should be considered [5]. The success rate of PCI for SCAD is lower than PCI for atherosclerotic disease and is associated with higher complications [13]. It is challenging due to the usage of multiple stents in long lesions; the wires may enter the false lumen, occlude the vessels, and further create an iatrogenic injury, which propagates the hematoma and results in the loss of distal-vessel patency. CABG is limited to those with high-risk anatomical lesions who have PCI failure, those with a large area of myocardial ischemia, and those who have been medically treated but are not sufficient to treat the ongoing ischemia [14]. Surgery is also recommended in patients with LMCA SCAD, but it is limited to the difficulty of finding the true lumen and is associated with mortality. Left-main lesions may also be treated by PCI stents [17]. The percutaneous approach in LMA SCAD is uncommon [12]. Proximal and distal progression may develop while advancing the guidewire through the true lumen [17]. Our patient had successful PCI in his left main coronary artery and no complications after the procedure. Revascularization improves the blood flow in the dissected vessels, but it doesn’t prevent the extension of dissection, and hospital readmission at 30 days may also happen [14].

## Conclusions

Acute coronary syndrome is a common cause of acute onset chest pain. Electrocardiography is a useful tool for diagnosis, risk stratification, and monitoring treatment response. Changes in the aVR lead may be caused by localized CAD and may be a sign of poor prognostic marker in ACS patients. SCAD is an important cause of ACS and may also manifest as acute chest pain. It may be diagnosed early by coronary angiography and, if indicated, it is well-treated by revascularization, either PCI or CABG.
